# Normalisation of airflow limitation in asthma: Post‐hoc analyses of TRIMARAN and TRIGGER

**DOI:** 10.1002/clt2.12145

**Published:** 2022-04-17

**Authors:** Alberto Papi, Dave Singh, J. Christian Virchow, G. Walter Canonica, Andrea Vele, George Georges

**Affiliations:** ^1^ Respiratory Medicine Unit University of Ferrara, University Hospital S. Anna Ferrara Italy; ^2^ Medicines Evaluation Unit The University of Manchester, Manchester University NHS Foundation Trust Manchester UK; ^3^ Departments for Pneumology/Internal Intensive Care Medicine Center for Internal Medicine, University Medicine Rostock Rostock Germany; ^4^ Center of Personalized Medicine: Asthma and Allergy Humanitas University and Research Hospital IRCCS Milan Italy; ^5^ Global Clinical Development Chiesi Farmaceutici SpA Parma Italy

**Keywords:** exacerbations, inhaled corticosteroid, inhaled triple therapy, long‐acting muscarinic antagonist, long‐acting beta2‐agonist

## Abstract

**Background:**

In asthma, persistent airflow limitation (PAL) is associated with poorer control, lung function decline and exacerbations. Using post‐hoc analyses we evaluated: the relationship between post‐salbutamol PAL at screening, airflow limitation (AL) during 52 weeks treatment with extrafine beclometasone dipropionate/formoterol fumarate/glycopyrronium (BDP/FF/G) versus BDP/FF and the risk of moderate/severe asthma exacerbations.

**Methods:**

TRIMARAN and TRIGGER were double‐blind studies comparing BDP/FF/G with BDP/FF (TRIMARAN medium‐dose ICS; TRIGGER high‐dose) in adults with uncontrolled asthma. Patients were subgrouped according to post‐salbutamol PAL status at screening, and AL over the 52‐week treatment period.

**Results:**

Most patients with post‐salbutamol PAL at screening had AL at all on‐treatment visits (TRIMARAN 62.8%; TRIGGER 66.8%). A significantly higher proportion of patients had normalised airflow on ≥1 follow‐up visit when receiving BDP/FF/G than BDP/FF (TRIMARAN 44.1 vs. 33.1% [*p* = 0.003]; TRIGGER 40.1 vs. 26.0% [*p* < 0.001]). In patients with post‐salbutamol PAL at screening and normalised AL at ≥1 follow‐up visit, exacerbation rates were 15% (*p* = 0.105) and 19% (*p* = 0.039) lower in TRIMARAN and TRIGGER versus those with AL on all visits. There was a trend to lower exacerbation rates in patients receiving BDP/FF/G than BDP/FF, particularly in patients in whom AL was normalised.

**Conclusion:**

In these analyses, AL in asthma was associated with an increased exacerbation incidence. Inhaled triple therapy with extrafine BDP/FF/G was more likely to normalise airflow, and was associated with a trend to a lower exacerbation rate than BDP/FF, particularly in the subgroup of patients in whom treatment was associated with airflow normalisation.

ClinicalTrials.gov: TRIMARAN, NCT02676076; TRIGGER, NCT02676089.

## 1 INTRODUCTION

One of the aims of asthma management is to minimise the development of persistent airflow limitation (PAL),^1^ usually defined as a forced expiratory volume in 1 s (FEV_1_) to forced vital capacity (FVC) ratio that does not normalise after administration of a short‐acting β_2_‐agonist. Although potentially a distinct phenotype,^2^ the risk factors associated with PAL are unclear. Indeed, one study has shown that a higher exacerbation rate could subsequently lead to the development of PAL,^3^ whereas a second study suggested that the risk factors included age, asthma duration and male sex, but not exacerbation history.^4^ Importantly, PAL could be a marker for corticosteroid resistance.^5^

The presence of PAL in patients with asthma is associated with increased bronchial inflammation and airway remodelling,^5^, ^6^, ^7^ poorer subsequent asthma control,^8^ and increased lung function decline.^9^ Furthermore, patients with asthma who have PAL are more likely to also have small airways dysfunction.^10^ However, whether PAL in asthma is a permanent condition, as it is in chronic obstructive pulmonary disease, or a potentially reversible state has never been properly tested. The ordered setting of a randomised controlled trial seems appropriate for such an assessment.

TRIMARAN and TRIGGER were two 52‐week clinical studies that recruited patients with asthma that was uncontrolled despite treatment with a fixed combination of a long‐acting β_2_‐agonist (LABA) and medium‐ (TRIMARAN) or high‐dose inhaled corticosteroid (ICS; TRIGGER), and pre‐bronchodilator FEV_1_ <80% of predicted normal, but no limitation on post‐bronchodilator FEV_1_ or FEV_1_/FVC ratio.^11^ As each study recruited more than 1000 patients, post‐hoc analyses provide an opportunity to evaluate the relationship between post‐salbutamol PAL at screening and the occurrence of asthma exacerbations over the subsequent 52 weeks. Furthermore, patients in both studies were randomised to either triple therapy with extrafine beclometasone dipropionate/formoterol fumarate/glycopyrronium (BDP/FF/G) or to the extrafine ICS/LABA combination BDP/FF. Overall in these studies, the addition of the long‐acting muscarinic antagonist (LAMA) component improved lung function and reduced the risk of asthma exacerbations.^11^ In previous post‐hoc analyses, we demonstrated that the efficacy of BDP/FF/G was more pronounced in the subgroup of patients with post‐salbutamol PAL at screening compared to the overall population in both studies, consistently providing statistically superior bronchodilator efficacy to BDP/FF.^12^ In addition, the effect of BDP/FF/G on moderate/severe exacerbations appeared greater in the PAL subset in TRIGGER. We therefore used data from TRIMARAN and TRIGGER to better understand the relationship between normalisation of airflow limitation (following maintenance therapy with triple therapy or ICS/LABA) and the risk of asthma exacerbations in the subgroup of patients who exhibited post‐salbutamol PAL at screening.

## 2 MATERIALS AND METHODS

### 2.1 Trial design and participants

The full design and inclusion/exclusion criteria of TRIMARAN and TRIGGER have been previously published.^11^ Both studies recruited patients aged 18–75 years, with a history of asthma for ≥1 year and diagnosed prior to the age of 40 years, pre‐bronchodilator FEV_1_ <80% predicted, and a change in FEV_1_ of >12% and >200 ml 10–15 min after inhaling salbutamol 400 µg. Patients had uncontrolled asthma (Asthma Control Questionnaire [ACQ]‐7 ≥1.5), a history of ≥1 exacerbation requiring treatment with systemic corticosteroids or an emergency department visit or in‐patient hospitalisation in the previous 12 months, and were receiving a stable ICS/LABA dose for ≥4 weeks prior to entry (TRIMARAN: medium ICS dose; TRIGGER: high ICS dose). All patients were non‐ or ex‐smokers; ex‐smokers with ≥10 pack‐years exposure or who stopped smoking ≤1 year prior to screening were excluded.

Patients who met all inclusion and no exclusion criteria at screening had their asthma maintenance therapy switched to extrafine BDP/FF 100/6 µg in TRIMARAN and 200/6 µg in TRIGGER, two inhalations twice daily (BID) via pressurised metred‐dose inhaler (pMDI) for a 2‐week open‐label run‐in period. At the end of the run‐in period, patients were randomised to either continue BDP/FF (100/6 µg in TRIMARAN, 200/6 µg in TRIGGER) or to receive extrafine BDP/FF/G (100/6/10 µg in TRIMARAN, 200/6/10 µg in TRIGGER), all two inhalations BID via pMDI. A third treatment group was included in TRIGGER: open‐label BDP/FF plus tiotropium in separate inhalers; these patients are not included in the current analyses, as this group was smaller than the other two treatment groups (patients were randomised into TRIGGER in a 2:2:1 ratio). Over the 52‐week treatment period, patients attended visits at baseline and after 4, 12, 26, 40, and 52 weeks at which data were collected from spirometry evaluations, with asthma exacerbations captured throughout the study.

All patients provided written informed consent prior to any study‐related procedure. The study was approved by the independent ethics committees at each institution, and was performed in accordance with the principles of the Declaration of Helsinki, and the International Conference on Harmonisation notes for guidance on Good Clinical Practice (ICH/CPMP/135/95). The studies are registered with ClinicalTrials.gov: TRIMARAN, NCT02676076; TRIGGER, NCT02676089.

### 2.2 Outcomes

These post‐hoc analyses focus on the rates of moderate‐to‐severe exacerbations in each study. Severe exacerbations were defined as asthma worsening requiring treatment with systemic corticosteroids for at least 3 days, whereas moderate exacerbations were episodes of asthma worsening that were self‐managed, defined in accordance with an American Thoracic Society/European Respiratory Society joint statement (one or more of: nocturnal awakenings due to asthma or increased daily symptoms; increased short‐acting β_2_‐agonist use; decline in FEV_1_ or peak expiratory flow; and/or emergency room/study site visit for asthma treatment not requiring systemic corticosteroids).^11^, ^13^

### 2.3 Statistical methods

The analyses include patients with post‐salbutamol (400 µg) FEV_1_/FVC ratio data available at the screening visit (corresponding to the peak bronchodilator effect) and with FEV_1_/FVC ratio data available at 3 h after administration of study drug (‘3 h post‐dose’, corresponding to the peak bronchodilator effect using BDP/FF/G or BDP/FF instead of salbutamol) on at least one post‐randomisation visit (i.e., from Week 0 to Week 52). Patients were subgrouped according to PAL status at screening, and to on‐treatment airflow limitation status over the 52‐week treatment period, as follows:

- PAL at screening: Patients were considered to have PAL if they had a FEV_1_/FVC ratio <0.7 10–15 min after administration of salbutamol.
- On‐treatment airflow limitation: Patients were considered to have airflow limitation if they had 3 h post‐dose FEV_1_/FVC ratio <0.7 at all available post‐randomisation visits.

The number of asthma exacerbations over the 52‐week treatment period comparing patients without versus with on‐treatment airflow limitation was analysed using a negative binomial model including on‐treatment airflow limitation, treatment, country and number of exacerbations in the previous year (1 or >1) as fixed effects, and log‐time on study as offset. The number of asthma exacerbations over the 52‐week treatment period comparing BDP/FF/G versus BDP/FF was analysed using a negative binomial model including treatment, country and number of exacerbations in the previous year (1 or >1) as fixed effects, and log‐time on study as offset. The proportion of patients who had a normalised FEV_1_/FVC ratio on ≥1 visit comparing BDP/FF/G versus BDP/FF was analysed using a logistic regression model including treatment, country and post‐salbutamol FEV_1_/FVC ratio at screening as covariates.

All analyses were replicated with the presence of airflow limitation defined using FEV_1_/FVC lower limit of normal (LLN) instead of the fixed‐ratio cut‐off 0.7, based on the Global Lung Function 2012 equations (taking into account patients' age, sex, height and race),^14^ and using the Global Lung Function Initiative calculator:^15^

- LLN‐derived PAL at screening: Patients were considered to have PAL if they had a FEV_1_/FVC ratio < LLN 10–15 min after administration of salbutamol.
- LLN‐derived on‐treatment airflow limitation: Patients were considered to have airflow limitation if they had 3 h post‐dose FEV_1_/FVC ratio < LLN at all available post‐randomisation visits.

All analyses were performed using SAS Version 9.4; the SAS procedure used to run the negative binomial models was PROC GENMOD.

## 3 RESULTS

**FIGURE 1:**
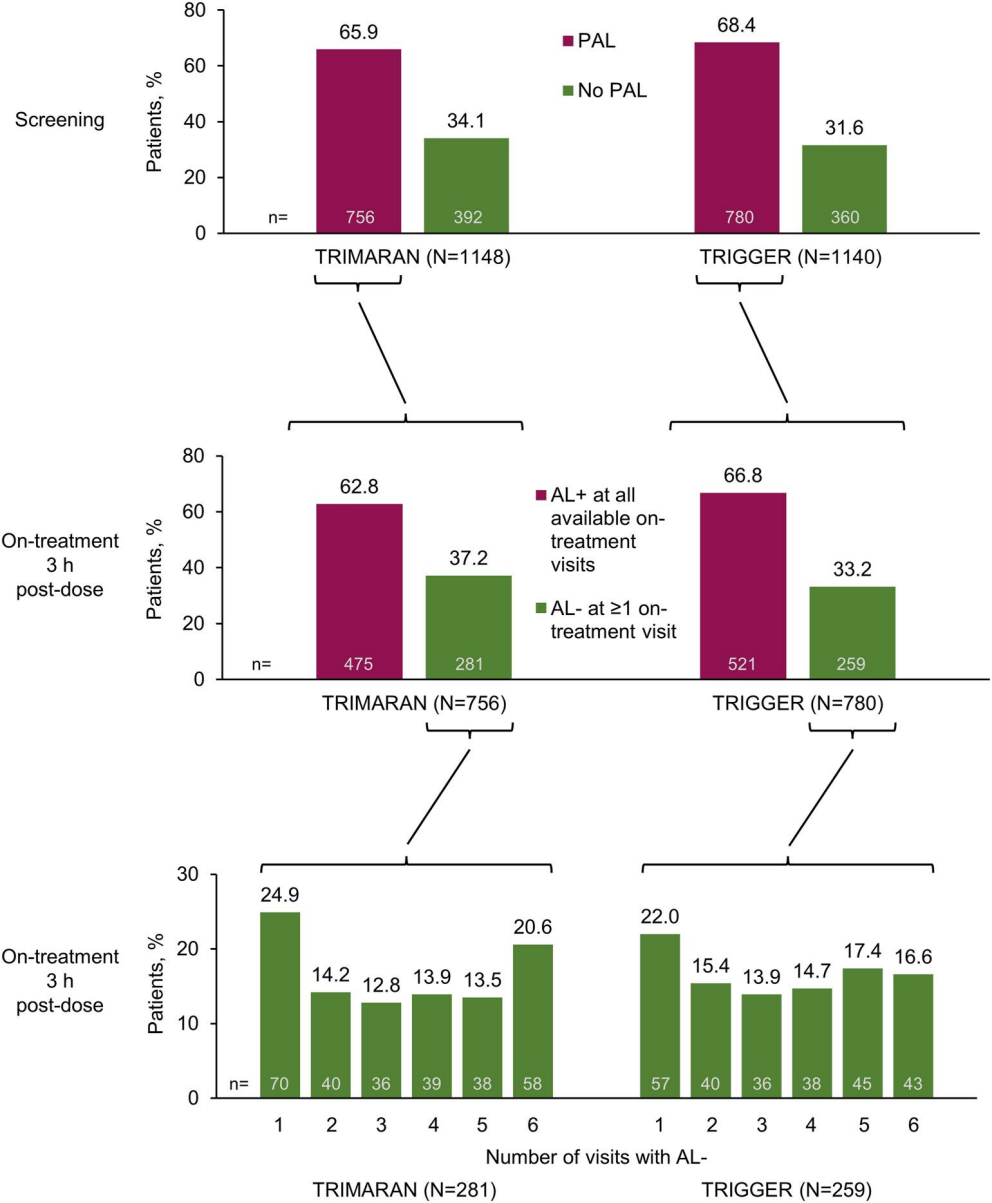
Patients in TRIMARAN and TRIGGER subgrouped by post‐salbutamol persistent airflow limitation (PAL) status at screening (top panel), and the subgroup of patients with post‐salbutamol PAL at screening then subgrouped by on‐treatment 3‐h post‐dose airflow limitation status during the studies. Screening: PAL, persistent airflow limitation, defined as post‐salbutamol FEV_1_/FVC <0.7; No PAL, no persistent airflow limitation, defined as post‐salbutamol FEV_1_/FVC ≥0.7. On‐treatment: AL+, airflow limitation, defined as all available post‐randomisation 3 h post‐dose FEV_1_/FVC <0.7; AL‐, normalisation of airflow limitation, defined as at least one post‐randomisation 3 h post‐dose FEV_1_/FVC ≥0.7

These analyses are based on data from 1148 patients in TRIMARAN and 1140 in TRIGGER who received randomised, double‐blinded treatment with BDP/FF/G or BDP/FF. Using the fixed‐ratio cut‐off, in TRIMARAN 756 (65.9%) patients had post‐salbutamol PAL at screening; in TRIGGER 780 (68.4%) patients met this criterion (Figure 1). Replacing the fixed ratio with the LLN cut‐off slightly reduced the proportion of patients with PAL at screening in both studies (59.8% and 61.8%; Figure S1).

The baseline characteristics of the patients subgrouped by post‐salbutamol PAL status at screening using the fixed‐ratio within each study are shown in Table 1. Compared to those without PAL, patients with post‐salbutamol PAL on entry to the studies were more likely to be male, ≥65 years of age, and prior smokers (current smokers were excluded from the studies). Importantly, the exacerbation history prior to enrolment in each study was similar in the PAL and no PAL subgroups (although, overall, patients in TRIGGER were more likely to have a history of >1 exacerbation than those in TRIMARAN). Results were similar using the LLN‐derived PAL status, with the exception of the age category: patients with post‐salbutamol PAL on entry were more likely to be <65 years of age (Table S1).

**TABLE 1:** Baseline characteristics of patients in TRIMARAN and TRIGGER, subgrouped by persistent airflow limitation status at screening

Parameter	TRIMARAN		TRIGGER	
	PAL at screening (*N* = 756)	No PAL at screening (*N* = 392)	PAL at screening (*N* = 780)	No PAL at screening (*N* = 360)
Sex, male, *n* (%)	333 (44.0)	109 (27.8)	351 (45.0)	104 (28.9)
Age, years, mean (SD)	54.0 (11.69)	49.8 (12.93)	55.2 (11.46)	50.2 (12.38)
Age group, *n* (%)				
<65 years	607 (80.3)	337 (86.0)	602 (77.2)	318 (88.3)
≥65 years	149 (19.7)	55 (14.0)	178 (22.8)	42 (11.7)
BMI, kg/m^2^, mean (SD)	27.8 (4.60)	28.3 (5.52)	28.4 (5.46)	28.7 (5.58)
BMI category, kg/m^2^, *n* (%)				
<25	226 (29.9)	118 (30.1)	217 (27.8)	95 (26.4)
25–<30	325 (43.0)	136 (34.7)	295 (37.8)	127 (35.3)
≥30	205 (27.1)	138 (35.2)	268 (34.4)	138 (38.3)
Smoking status, *n* (%)				
Ex‐smoker	127 (16.8)	41 (10.5)	126 (16.2)	37 (10.3)
Non‐smoker	629 (83.2)	351 (89.5)	654 (83.8)	323 (89.7)
ACQ‐5 at screening, mean (SD)	2.4 (0.72)	2.4 (0.69)	2.6 (0.73)	2.5 (0.63)
ACQ‐7 at screening, mean (SD)	2.6 (0.61)	2.5 (0.56)	2.9 (0.63)	2.6 (0.54)
Pre‐salbutamol FEV_1_% predicted, mean (SD)	52.4 (12.07)	61.2 (9.94)	48.0 (12.98)	60.0 (10.61)
Pre‐salbutamol FEV_1_/FVC, mean (SD)	0.55 (0.09)	0.70 (0.09)	0.53 (0.10)	0.71 (0.09)
Post‐salbutamol FEV_1_/FVC, mean (SD)	0.59 (0.08)	0.77 (0.05)	0.57 (0.10)	0.76 (0.05)
Asthma exacerbations in previous year, *n* (%)				
1	629 (83.2)	316 (80.6)	607 (77.8)	282 (78.3)
>1	127 (16.8)	76 (19.4)	173 (22.2)	78 (21.7)

*Note*: PAL, persistent airflow limitation, defined as post‐salbutamol FEV_1_/FVC < 0.7; No PAL, no persistent airflow limitation, defined as post‐salbutamol FEV_1_/FVC ≥ 0.7.

Abbreviations: ACQ, Asthma Control Questionnaire; BMI, body‐mass index.

### 3.1 Relationship between post‐salbutamol PAL at screening and on‐treatment airflow limitation

The majority of patients with post‐salbutamol PAL at screening also had airflow limitation at 3 h post‐dose on all visits for the duration of the two studies, both using the fixed‐ratio cut‐off (475 of the 756 patients [62.8%] in TRIMARAN, and 521 of the 780 patients [66.8%] in TRIGGER; Figure 1) and the LLN cut‐off (57.2% and 61.8%; Figure S1). Patients who had post‐salbutamol PAL at screening that was then normalised by study treatment (i.e., 3 h post‐dose FEV_1_/FVC ratio ≥0.7 on at least one study visit) were more likely to be female and <65 years of age in both studies and using both cut‐offs (i.e., 0.7 or LLN; Table 2 and Table S2). In these patients, the number of visits at which airflow was normalised (i.e., 3 h post‐dose FEV_1_/FVC ≥0.7) ranged from one to six, with 20.6% of patients in TRIMARAN and 16.6% in TRIGGER having normalisation at all six visits using the fixed‐ratio cut‐off (Figure 1). Using the LLN cut‐off, 22.8% and 21.2% of patients had airflow normalisation at all six visits (Figure S1).

**TABLE 2:** Baseline characteristics of the subgroup of patients with post‐salbutamol persistent airflow limitation at screening, subgrouped by on‐treatment 3‐h post‐dose airflow limitation status

Parameter	TRIMARAN		TRIGGER	
	PAL at screening (*N* = 756)		PAL at screening (*N* = 780)	
	AL + at all available visits (*N* = 475)	AL– at ≥1 visit (*N* = 281)	AL + at all available visits (*N* = 521)	AL– at ≥1 visit (*N* = 259)
Sex, male, *n* (%)	239 (50.3)	94 (33.5)	279 (53.6)	72 (27.8)
Age, years, mean (SD)	55.9 (10.58)	50.7 (12.71)	56.8 (10.72)	52.0 (12.22)
Age group, *n* (%)				
<65 years	367 (77.3)	240 (85.4)	386 (74.1)	216 (83.4)
≥65 years	108 (22.7)	41 (14.6)	135 (25.9)	43 (16.6)
BMI, kg/m^2^, mean (SD)	27.6 (4.46)	28.0 (4.83)	28.2 (5.19)	28.9 (5.96)
BMI category, kg/m^2^, *n* (%)				
<25	138 (29.1)	88 (31.3)	149 (28.6)	68 (26.3)
25–<30	218 (45.9)	107 (38.1)	198 (38.0)	97 (37.5)
≥30	119 (25.1)	86 (30.6)	174 (33.4)	94 (36.3)
Smoking status, *n* (%)				
Ex‐smoker	84 (17.7)	43 (15.3)	93 (17.9)	33 (12.7)
Non‐smoker	391 (82.3)	238 (84.7)	428 (82.1)	226 (87.3)
ACQ‐5 at screening, mean (SD)	2.3 (0.69)	2.4 (0.77)	2.6 (0.72)	2.6 (0.75)
ACQ‐7 at screening, mean (SD)	2.6 (0.60)	2.6 (0.63)	2.9 (0.61)	2.8 (0.66)
Pre‐salbutamol FEV_1_% predicted, mean (SD)	49.8 (12.10)	56.9 (10.61)	45.0 (12.59)	54.2 (11.49)
Pre‐salbutamol FEV_1_/FVC, mean (SD)	0.52 (0.08)	0.61 (0.08)	0.50 (0.09)	0.60 (0.08)
Post‐salbutamol FEV_1_/FVC, mean (SD)	0.56 (0.08)	0.65 (0.05)	0.53 (0.09)	0.64 (0.05)
Asthma exacerbations in previous year, *n* (%)				
1	400 (84.2)	229 (81.5)	402 (77.2)	205 (79.2)
>1	75 (15.8)	52 (18.5)	119 (22.8)	54 (20.8)

*Note*: Screening: PAL, airflow limitation, defined as post‐salbutamol FEV_1_/FVC < 0.7. On‐treatment: AL+, airflow limitation, defined as all available post‐randomisation 3 h post‐dose FEV_1_/FVC < 0.7; AL‐, normalisation of airflow limitation, defined as at least one post‐randomisation 3 h post‐dose FEV_1_/FVC ≥ 0.7.

Abbreviations: ACQ, Asthma Control Questionnaire; BMI, body‐mass index.

### 3.2 Relationship between on‐treatment airflow limitation and the occurrence of moderate/severe asthma exacerbations

**FIGURE 2:**
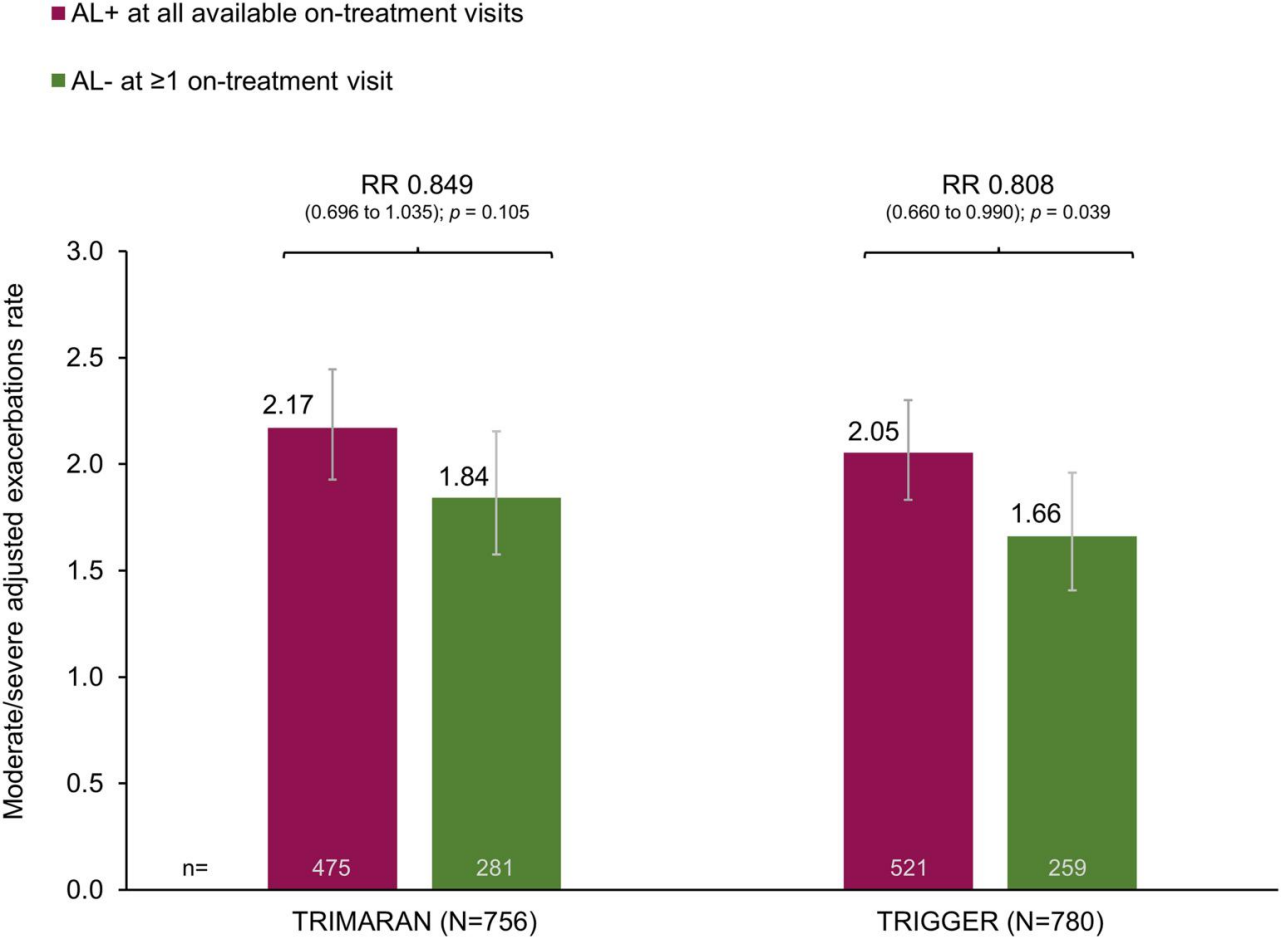
Adjusted rate of moderate/severe exacerbations in the subgroup of patients with post‐salbutamol PAL at screening, comparing patients by on‐treatment 3‐h post‐dose airflow limitation status. Data plotted are adjusted exacerbation rate and 95% confidence interval comparing patients without versus with on‐treatment airflow limitation. AL+, airflow limitation, defined as all available post‐randomisation 3 h post‐dose FEV_1_/FVC <0.7; AL‐, normalisation of airflow limitation, defined as at least one post‐randomisation 3 h post‐dose FEV_1_/FVC ≥0.7. PAL, persistent airflow limitation, defined as post‐salbutamol FEV_1_/FVC <0.7 at screening. RR, rate ratio (95% confidence interval)

The rates of on‐treatment moderate/severe exacerbations were analysed in the subgroup of patients with post‐salbutamol PAL at screening, regardless of the treatment received. In both studies, the rate of exacerbations was lower in patients who had normalised FEV_1_/FVC at 3 h post‐dose on ≥1 on‐treatment visit, with rates using the fixed‐ratio cut‐off that were 15% (*p* = 0.105) and 19% (*p* = 0.039) lower than in those who had airflow limitation at all on‐treatment visits in TRIMARAN and TRIGGER, respectively (Figure 2). The difference was more marked when using the LLN airflow limitation cut‐off, with rate reductions of 21% (*p* = 0.025) and 25% (*p* = 0.006), respectively (Figure S2).

### 3.3 Influence of study treatment on normalisation of airflow limitation

**FIGURE 3:**
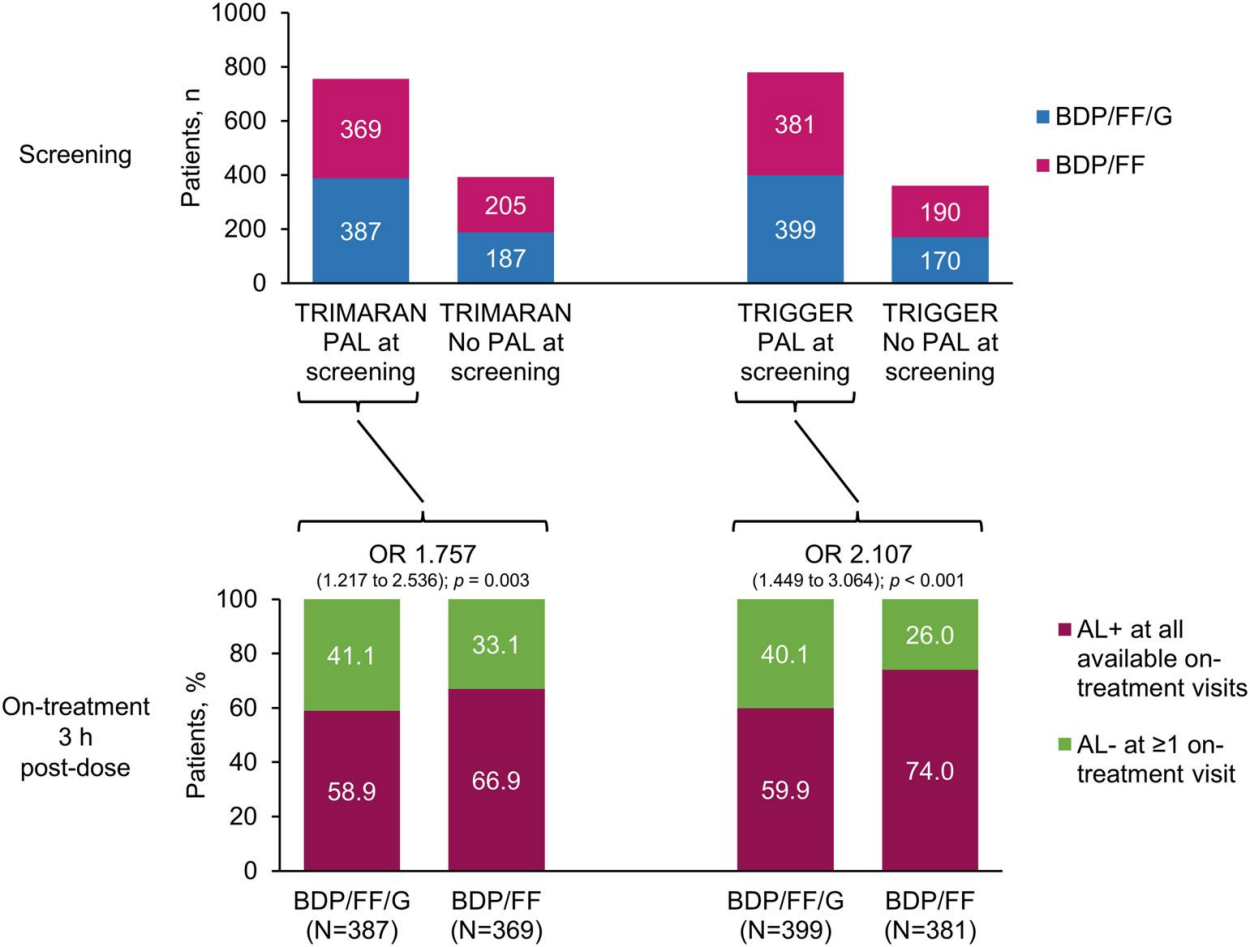
Relationship between study treatment received and normalisation of airflow limitation. OR, odds ratio for the proportion of patients with AL– at ≥1 on‐treatment visit, comparing BDP/FF/G versus BDP/FF. Screening: PAL, persistent airflow limitation, defined as post‐salbutamol FEV_1_/FVC <0.7; No PAL, no persistent airflow limitation, defined as post‐salbutamol FEV_1_/FVC ≥0.7. On‐treatment: AL+, airflow limitation, defined as all available post‐randomisation 3 h post‐dose FEV_1_/FVC <0.7; AL‐, normalisation of airflow limitation, defined as at least one post‐randomisation 3 h post‐dose FEV_1_/FVC ≥0.7. BDP, beclometasone dipropionate; FF, formoterol fumarate; G, glycopyrronium

There was no imbalance between treatment arms in the proportion of patients with post‐salbutamol PAL at screening using either airflow limitation cut‐off (Figure 3 and Figure S3). However, during the study, a significantly higher proportion of patients had normalised FEV_1_/FVC ratio on ≥1 visit when receiving BDP/FF/G compared to BDP/FF (fixed ratio: 41.1% vs. 33.1% in TRIMARAN [*p* = 0.003] and 40.1% vs. 26.0% in TRIGGER [*p* < 0.001]; LLN: 48.9% vs. 36.1% [*p* < 0.001] and 43.1% vs. 33.3% [*p* = 0.005]).

### 3.4 Relationship between study treatment and the occurrence of moderate/severe asthma exacerbations

**FIGURE 4:**
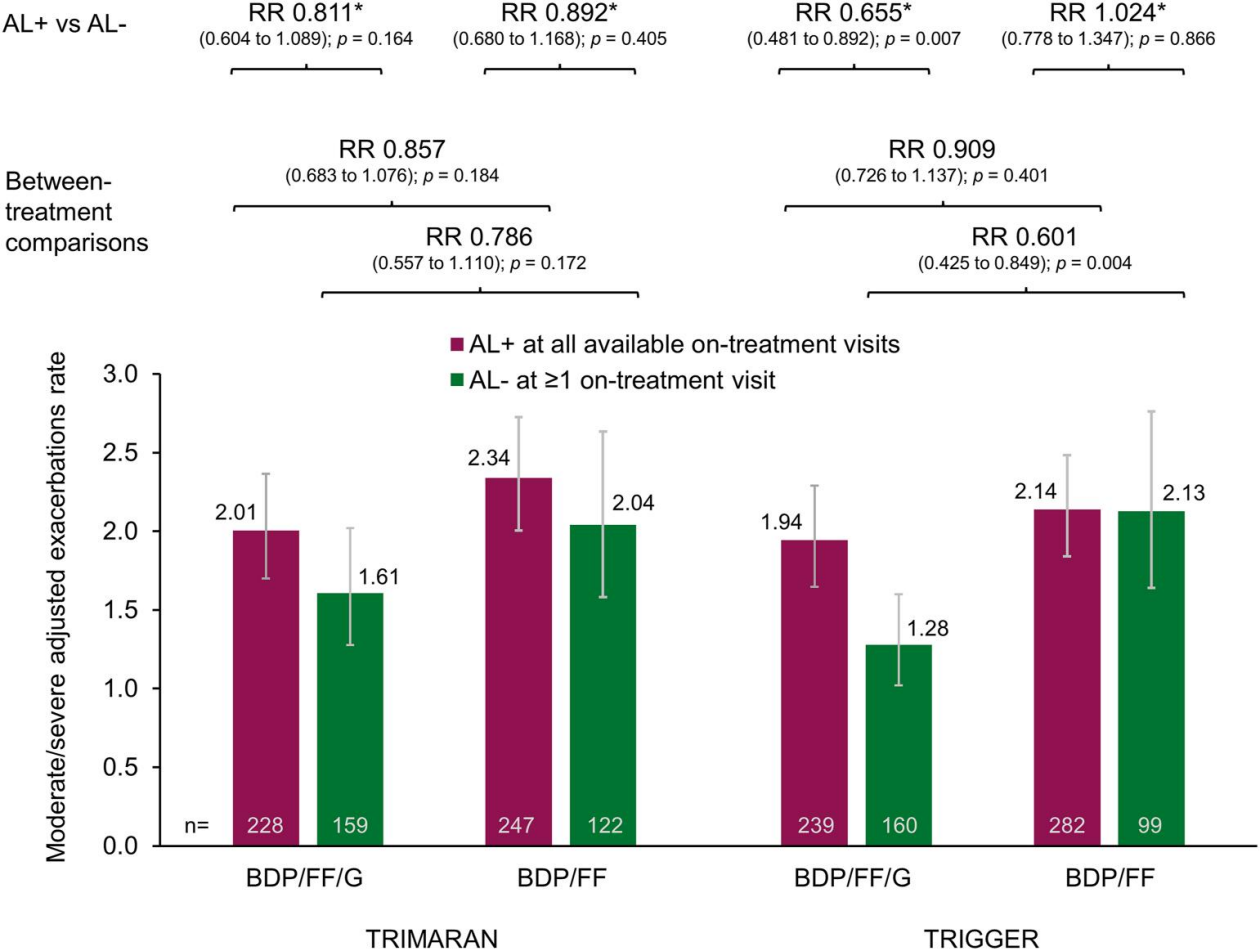
Adjusted rate of moderate/severe exacerbations in the subgroup of patients with post‐salbutamol PAL at screening, with patients subgrouped by on‐treatment 3‐h post‐dose airflow limitation status and by treatment. Data plotted are adjusted exacerbation rate and 95% confidence interval from the statistical model subgrouping patients by on‐treatment AL and comparing BDP/FF/G versus BDP/FF (the rates from the statistical model subgrouping patients by the treatment received and comparing patients without vs. with on‐treatment airflow limitation were 2.02, 1.64, 2.32, 2.07, 2.02, 1.32, 2.10, and 2.15). AL+, airflow limitation, defined as all available post‐randomisation 3 h post‐dose FEV_1_/FVC <0.7; AL‐, normalisation of airflow limitation, defined as at least one post‐randomisation 3 h post‐dose FEV_1_/FVC ≥0.7. PAL, persistent airflow limitation, defined as post‐salbutamol FEV_1_/FVC <0.7 at screening. RR, rate ratio (95% confidence interval); BDP, beclometasone dipropionate; FF, formoterol fumarate; G, glycopyrronium

The rates of on‐treatment moderate/severe exacerbations during the studies were analysed in the subgroup of patients with post‐salbutamol PAL at screening. In patients receiving BDP/FF/G, the rate of moderate/severe exacerbations was lower in both studies in patients who had normalised FEV_1_/FVC at ≥1 on‐treatment visit than in those who continued to experience airflow limitation, with reductions of 19% (*p* = 0.164) and 34% (*p* = 0.007) in TRIMARAN and TRIGGER, respectively (Figure 4). For patients receiving BDP/FF, this was only the case in TRIMARAN (with an 11% reduction; *p* = 0.405); the rates in TRIGGER were the same in the two airflow limitation subgroups. Using the LLN cut‐off, the rate reductions in patients with normalised airflow (i.e., ≥LLN) versus those who continued to experience airflow limitation were generally larger than when using the fixed‐ratio definition, with reductions of 23% (*p* = 0.063) and 31% (*p* = 0.017) for patients receiving BDP/FF/G in TRIMARAN and TRIGGER, respectively, and 12% (*p* = 0.363) and 17% (*p* = 0.172), respectively, for those receiving BDP/FF (Figure S4). In both studies there was a general trend to lower exacerbation rates in patients receiving BDP/FF/G than BDP/FF, with the most relevant result in TRIGGER in the subgroup of patients with normalised FEV_1_/FVC at ≥1 visit, where the reduction was statistically significant (40% reduction when receiving BDP/FF/G vs. BDP/FF; *p* = 0.004).

## 4 DISCUSSION

In these analyses using data from two large, randomised clinical trials, we first confirmed that there is a population of patients with asthma who, despite having PAL at screening (measured 10–15 min post‐salbutamol), experience normalisation of airflow (i.e., FEV_1_/FVC ≥0.7 measured 3 h post‐dose) when receiving ICS/LABA or ICS/LABA/LAMA maintenance therapy. This suggests that post‐salbutamol PAL assessed at a single timepoint does not necessarily imply that an individual has fixed airflow limitation, but rather that airflow limitation can potentially be normalised by appropriate treatment; our analyses therefore question whether airflow limitation (in particular post‐salbutamol PAL) is a stable phenotype. Importantly, we demonstrated that normalisation of airflow limitation is an important treatment goal, since (regardless of the treatment received) these patients were overall at lower risk of on‐treatment asthma exacerbations than those who demonstrated airflow limitation at all available visits while on treatment.

The populations that we recruited into the two studies all had asthma that was uncontrolled despite receiving ICS/LABA for at least 4 weeks prior to entry (and almost all patients had been receiving ICS/LABA for at least 3 months^11^). Given we excluded patients who were also receiving a LAMA, a biologic, or other forms of asthma maintenance therapy,^11^ those we studied are, according to treatment strategy documents such as the Global Initiative for Asthma, candidates for inhaled triple ICS/LABA/LAMA therapy.^1^ In the overall analyses of TRIMARAN and TRIGGER, we demonstrated that the addition of a LAMA to ICS/LABA therapy improved lung function and reduced exacerbations.^11^ The current analyses suggest that part of this exacerbation benefit could be due to normalisation of airflow, with a significantly higher proportion having normalisation of airflow at one or more visits when receiving BDP/FF/G compared to BDP/FF. Furthermore, there was a general trend to lower on‐treatment exacerbation rates in patients receiving BDP/FF/G than BDP/FF, particularly in those in whom study treatment was associated with airflow normalisation. These results reinforce the benefit of adding a LAMA in patients whose asthma is uncontrolled on ICS/LABA.

Our results confirm that post‐salbutamol PAL is present in a substantial proportion of patients with asthma receiving ICS/LABA (approximately two‐thirds of the patients in each study at screening). Although such airflow limitation could be targeted with biologic therapy or systemic corticosteroids,^16^, ^17^, ^18^, ^19^ given concerns over cost or side effects, respectively, our results also suggest that a trial with inhaled triple therapy first would be appropriate. That a proportion of patients still had airflow limitation despite inhaled triple therapy could potentially be the result of lingering inflammation, or irreversible airway narrowing due to airway wall remodelling.^20^ Evidence suggests that such remodelling may result from repeated bronchoconstriction, rather from inflammation.^21^ An appropriate treatment strategy for a patient with PAL despite ICS/LABA could therefore be to escalate promptly to triple therapy, although additional research is required to determine whether this prevents the development of irreversible airflow limitation.

Post‐hoc, subgroup analyses such as those we present here are not formally statistically powered (due to the smaller number of patients analysed compared to the overall TRIMARAN and TRIGGER populations), and so are also prone to type II errors (or false negatives). This is especially possible when the endpoint of interest has a low incidence (as can be the case for exacerbations). Although we present the rate ratios and associated *p* values in many of these analyses, we would argue that a lack of statistical significance does not necessarily indicate a lack of relevant difference. In addition, given post‐hoc analyses are typically not adjusted for multiplicity, findings in such analyses that have *p* values lower than the usual threshold (i.e., <0.05) could theoretically also be linked to type I error (or false positives). We have therefore focused on the consistency of findings between TRIMARAN and TRIGGER when drawing conclusions (one of the reasons that we did not pool the data).

It is challenging to compare the results of our analyses with those from other studies, partly due to differences in the definitions of airflow limitation and exacerbations. However, our analyses confirm and expand on results of a previous study, in which baseline PAL (post‐salbutamol FEV_1_/FVC <0.7) was associated with a higher rate of asthma exacerbations than in patients with asthma who had reversible airflow limitation (1.41 vs. 0.53; *p* < 0.01).^9^ Similarly, in the “Difficult Asthma Study”, patients with baseline PAL (again post‐salbutamol FEV_1_/FVC <0.7) had a significantly higher rate of exacerbations at baseline than those whose airflow normalised following salbutamol administration (1 vs. 0 [the authors express the rates only as complete integers]; *p* < 0.05).^3^ Furthermore, when airflow limitation was assessed at a follow‐up visit at least 3 years after baseline, the exacerbation rate in the prior year was significantly higher in patients with airflow limitation at both visits (i.e., at baseline and at follow‐up) than in those with no airflow limitation at both visits (1 vs. 0; *p* < 0.01). An important difference from the current analyses, however, was that none of the participants in the Difficult Asthma Study with baseline PAL had normalised airflow at the follow‐up visit. Furthermore, neither of these prior analyses examined the effect of treatment. A post‐hoc analysis of data from two 12‐week studies did examine the effect of treatment—but only according to airflow limitation at baseline.^8^ Patients with baseline PAL (with two sets of analyses, FEV_1_/FVC < LLN, and FEV_1_/FVC <0.70) had a greater relative benefit from ICS/LABA versus ICS or LABA monotherapy than patients with reversible airflow limitation. In a second publication, the authors used data from one of these studies to evaluate the relationship between on‐treatment airflow limitation and treatment effect,^22^ although since the study was relatively small (386 patients) and only 12 weeks in duration, the effect on exacerbations was not analysed. Our analyses build on these various prior analyses, given we were able to use data from a full 1‐year follow‐up of a large number of patients who demonstrated reversibility at screening, who attended regular visits and were highly adherent to therapy, and with pre‐ and post‐treatment lung function and exacerbation occurrence collected throughout the follow‐up period.

In the initial analyses, we defined airflow limitation on the basis of a fixed FEV_1_/FVC ratio of 0.7. This can introduce an age bias, with older individuals more likely to meet the criterion.^23^ The use of a LLN can avoid such a limitation, although obviously adds complexity to the practical implementation of the findings. We therefore replicated the analyses using an LLN‐derived airflow limitation definition (i.e., <LLN). Although this impacted some of the data (most notably the proportion of patients aged ≥65 years who met the screening PAL definition, which was reduced from 19.7% to 22.8% in TRIMARAN and TRIGGER, respectively, using the fixed‐ratio cut‐off to 16.3% and 19.7%, respectively, using the LLN cut‐off), the overall results were consistent with the fixed‐ratio definition results. This is consistent with one of the prior studies that used both definitions to identify airflow obstruction at baseline, in which the treatment outcomes were generally similar for the two definitions.^8^ We therefore chose to focus the manuscript on the fixed‐ratio data, since at an individual patient level (when the focus is on changes over time), these results are easier to interpret than is the case for more intangible (and population‐dependent) LLN values.

There are some limitations to these analyses. The main limitation is that they are unpowered and not pre‐planned, subgroup comparisons. Of course, as with all such subgroup analyses, the results need to be confirmed in suitably designed prospective clinical trials. In addition, we analysed the occurrence of moderate and severe asthma exacerbations, rather than focussing on the arguably more clinically relevant severe exacerbations. This was because few patients experienced severe exacerbations during each study, and so subgroup comparisons suffer even more from a lack of power (even if data from the two studies were pooled). Our analyses focus on patients who had post‐salbutamol PAL at screening that was subsequently normalised by maintenance therapy, and did not include patients who had no PAL at screening but then developed airflow limitation during treatment. This latter population was very small (10% of patients in TRIMARAN and 6% in TRIGGER) and would therefore be insufficient to draw robust conclusions. Furthermore, we do not know whether patients who experienced airflow limitation at all available on‐treatment visits when receiving ICS/LABA or triple therapy would continue to have airflow limitation if they received additional doses of salbutamol or more aggressive treatment (such as oral corticosteroids). In addition, defining airflow limitation using salbutamol‐induced change after 10–15 min could be argued to be quite different from defining airflow limitation during the course of the study using a 3 h post‐dose response to BDP/FF or BDP/FF/G. Factors such as differences in time of the day, time to peak or plateau bronchodilation, and regression to the mean (since recruited patients had pre‐bronchodilator FEV_1_ <80% predicted) should also be considered. However, this scenario mimics treatment administration in the real world making this analysis even more relevant.

In conclusion, in patients with asthma who were receiving medium‐ or high‐dose ICS/LABA or ICS/LABA/LAMA, airflow limitation was associated with a general trend to an increased incidence of moderate‐to‐severe exacerbations. Treatment with extrafine BDP/FF/G was more likely to normalise airflow limitation and tended to be associated with a lower exacerbation rate than treatment with BDP/FF, with the most relevant results in the subgroup of patients in whom treatment was associated with normalisation of airflow limitation.

## CONFLICT OF INTEREST

Alberto Papi reports grants, personal fees, non‐financial support and payment for advisory board membership, consultancy, payment for lectures, grants for research, and travel expenses reimbursement from Chiesi, AstraZeneca, GlaxoSmithKline, Boehringer Ingelheim, Mundipharma and TEVA, and personal fees and non‐financial support from Menarini, Novartis, Zambon and Sanofi, all outside the submitted work. Dave Singh reports personal fees from Chiesi during the conduct of the studies. Outside the submitted work, he reports personal fees from AstraZeneca, Boehringer Ingelheim, Chiesi, Cipla, Genentech, GlaxoSmithKline, Glenmark, Menarini, Mundipharma, Novartis, Peptinnovate, Pfizer, Pulmatrix, Theravance, and Verona. J. Christian Virchow reports personal fees from Chiesi during the conduct of the studies. In the past J. Christian Virchow has lectured and received honoraria from AstraZeneca, Avontec, Bayer, Bencard, Bionorica, Boehringer‐Ingelheim, Chiesi, Essex/Schering‐Plough, GSK, Janssen‐Cilag, Leti, MEDA, Merck, MSD, Mundipharma, Novartis, Nycomed/Altana, Pfizer, Revotar, Sanofi/Regeneron, Sandoz‐Hexal, Stallergens, TEVA, UCB/Schwarz‐Pharma, Zydus/Cadila and possibly others, and participated in advisory boards and received honoraria from Avontec, Boehringer‐Ingelheim, Chiesi, Essex/Schering‐Plough, GSK, Janssen‐Cilag, MEDA, MSD, Mundipharma, Novartis, Paul‐Ehrlich Institut, Regeneron, Revotar, Roche, Sanofi‐Aventis, Sanofi/Regeneron, Sandoz‐Hexal, TEVA, UCB/Schwarz‐Pharma and possibly others, and received funding for research from Deutsche Forschungsgesellschaft, Land Mecklenburg‐Vorpommern, GSK, and MSD, and has advised the Bemeinsame Bundesausschuss (GBA). G. Walter Canonica reports personal fees from A. Menarini, Alk‐Abello, Allergy Therapeutics, AstraZeneca‐Medimmune, Boehringer Ingelheim, Chiesi Farmaceutici, Genentech, Guidotti‐Malesci, Glaxo Smith Kline, Hal Allergy, Merck Sharp & Dome, Mundipharma, Novartis, Orion, Sanofi‐Aventis, Sanofi Genzyme/Regeneron, Stallergenes‐Greer, Uriach Pharma, Teva, Valeas, ViforPharma, all outside the submitted work. Andrea Vele is an employee of Chiesi, the sponsor of the studies. George Georges is an employee of Chiesi USA, Inc.

## AUTHOR CONTRIBUTIONS

**Alberto Papi:** Conceptualization; Equal, Methodology; Equal, Visualization; Equal, Writing ‐ review & editing; Equal, **Dave Singh:** Conceptualization; Equal, Methodology; Equal, Visualization; Equal, Writing ‐ review & editing; **Equal, J. Virchow:** Conceptualization; Equal, Methodology; Equal, Visualization; Equal, Writing ‐ review & editing; Equal, **Giorgio Walter Canonica:** Conceptualization; Equal, Methodology; Equal, Visualization; Equal, Writing ‐ review & editing; **Equal, Andrea Vele:** Conceptualization; Equal, Formal analysis; Lead, Methodology; Equal, Visualization; Equal, Writing ‐ review & editing; Equal, **George Georges:** Conceptualization; Equal, Methodology; Equal, Visualization; Equal, Writing ‐ review & editing; Equal.

## Supporting information

Supplementary Material

## Data Availability

Chiesi commits to sharing with qualified scientific and medical researchers, conducting legitimate research, the anonymised patient‐level and study‐level data, the clinical protocol and the full clinical study report of Chiesi Farmaceutici SpA‐sponsored interventional clinical trials in patients for medicines and indications approved by the European Medicines Agency and/or the US Food and Drug Administration after 1st January 2015, following the approval of any received research proposal and the signature of a Data Sharing Agreement. Chiesi provides access to clinical trial information consistently with the principle of safeguarding commercially confidential information and patient privacy. Other information on Chiesi's data sharing commitment, access and research request's approval process are available in the Clinical Trial Transparency section of http://www.chiesi.com/en/research‐and‐development.
