# Supplementary material for: Normalisation of airflow limitation in asthma: Post‐hoc analyses of TRIMARAN and TRIGGER
Source: Clin Transl Allergy. 2022 Apr 17;12(4):e12145. doi: 10.1002/clt2.12145 (PMC9014197; doi:10.1002/clt2.12145)
Supplement: Supplementary file 1 — Supplementary Material [file CLT2-12-e12145-s001.docx]

# Normalisation of airflow limitation in asthma: *Post-hoc* analyses of TRIMARAN and TRIGGER

Alberto Papi, Dave Singh, J. Christian Virchow, G. Walter Canonica, Andrea Vele, George Georges

# Supplement

# Tables

Supplementary Table 1. Baseline characteristics of patients in TRIMARAN and TRIGGER, subgrouped by LLN-derived PAL status at screening.

| **Parameter** | **TRIMARAN** | | **TRIGGER** | |
| --- | --- | --- | --- | --- |
|  | **PAL (<LLN) (N=687)** | **No PAL (≥LLN) (N=461)** | **PAL (<LLN) (N=704)** | **No PAL ≥LLN (N=436)** |
| Sex, male, n (%) | 297 (43.2) | 145 (31.5) | 315 (44.7) | 140 (32.1) |
| Age, years, mean (SD) | 52.4 (12.00) | 52.8 (12.69) | 53.7 (11.91) | 53.4 (12.10) |
| Age group, n (%) |  |  |  |  |
| <65 years | 575 (83.7) | 369 (80.0) | 565 (80.3) | 355 (81.4) |
| ≥65 years | 112 (16.3) | 92 (20.0) | 139 (19.7) | 82 (18.6) |
| BMI, kg/m^2^, mean (SD) | 27.5 (4.56) | 28.5 (5.40) | 28.2 (5.44) | 29.0 (5.56) |
| BMI category, kg/m^2^, n (%) |  |  |  |  |
| <25 | 218 (31.7) | 126 (27.3) | 206 (29.3) | 106 (24.3) |
| 25–<30 | 179 (26.1) | 164 (35.6) | 225 (32.0) | 181 (41.5) |
| ≥30 | 290 (42.2) | 171 (37.1) | 273 (38.8) | 149 (34.2) |
| Smoking status, n (%) |  |  |  |  |
| Ex-smoker | 109 (15.9) | 59 (12.8) | 113 (16.1) | 50 (11.5) |
| Non-smoker | 578 (84.1) | 402 (87.2) | 591 (83.9) | 386 (88.5) |
| ACQ-5 at screening, mean (SD) | 2.1 (0.64) | 2.1 (0.60) | 2.3 (0.65) | 2.2 (0.56) |
| ACQ-7 at screening, mean (SD) | 2.3 (0.55) | 2.3 (0.48) | 2.5 (0.56) | 2.4 (0.48) |
| Pre-salbutamol FEV_1_ % predicted, mean (SD) | 51.5 (11.83) | 61.4 (9.95) | 47.1 (12.89) | 59.5 (10.58) |
| Pre-salbutamol FEV_1_/FVC, mean (SD) | 0.54 (0.09) | 0.69 (0.09) | 0.52 (0.10) | 0.70 (0.09) |
| Post-salbutamol FEV_1_/FVC, mean (SD) | 0.58 (0.08) | 0.75 (0.06) | 0.56 (0.09) | 0.75 (0.06) |
| Asthma exacerbations in previous year, n (%) |  |  |  |  |
| 1 | 568 (82.7) | 377 (81.8) | 542 (77.0) | 347 (79.6) |
| >1 | 119 (17.3) | 84 (18.2) | 162 (23.0) | 89 (20.4) |

PAL, persistent airflow limitation, defined as post-salbutamol FEV_1_/FVC <LLN; No PAL, no persistent airflow limitation, defined as post-salbutamol FEV_1_/FVC ≥LLN. LLN, lower limit of normal; BMI, body-mass index; ACQ, Asthma Control Questionnaire.

Supplementary Table 2. Baseline characteristics of the subgroup of patients with post-salbutamol LLN-derived PAL at screening, subgrouped by on-treatment 3-h post-dose LLN-derived airflow limitation status.

| **Parameter** | **TRIMARAN** | | **TRIGGER** | |
| --- | --- | --- | --- | --- |
|  | **PAL (<LLN) at screening (N=687)** | | **PAL (<LLN) at screening (N=704)** | |
|  | **AL+ (<LLN) at all available visits (N=393)** | **AL– (≥LLN) at  ≥1 visit (N=294)** | **AL+ (<LLN) at all available visits (N=435)** | **AL– (≥LLN) at  ≥1 visit (N=269)** |
| Sex, male, n (%) | 196 (49.9) | 101 (34.4) | 225 (51.7) | 90 (33.5) |
| Age, years, mean (SD) | 53.3 (11.47) | 51.3 (12.61) | 54.5 (11.69) | 52.5 (12.19) |
| Age group, n (%) |  |  |  |  |
| <65 years | 326 (83.0) | 249 (84.7) | 343 (78.9) | 222 (82.5) |
| ≥65 years | 67 (17.0) | 45 (15.3) | 92 (21.1) | 47 (17.5) |
| BMI, kg/m^2^, mean (SD) | 27.4 (4.50) | 27.7 (4.64) | 28.0 (5.49) | 28.5 (5.36) |
| BMI category, kg/m^2^, n (%) |  |  |  |  |
| <25 | 122 (31.0) | 96 (32.7) | 137 (31.5) | 69 (25.7) |
| 25–<30 | 97 (24.7) | 82 (27.9) | 137 (31.5) | 88 (32.7) |
| ≥30 | 174 (44.3) | 116 (39.5) | 161 (37.0) | 112 (41.6) |
| Smoking status, n (%) |  |  |  |  |
| Ex-smoker | 73 (18.6) | 36 (12.2) | 81 (18.6) | 32 (11.9) |
| Non-smoker | 320 (81.4) | 258 (87.8) | 354 (81.4) | 237 (88.1) |
| ACQ-5 at screening, mean (SD) | 2.1 (0.67) | 2.1 (0.61) | 2.3 (0.67) | 2.3 (0.62) |
| ACQ-7 at screening, mean (SD) | 2.4 (0.57) | 2.3 (0.51) | 2.5 (0.58) | 2.4 (0.54) |
| Pre-salbutamol FEV_1_ % predicted, mean (SD) | 48.3 (11.81) | 55.7 (10.48) | 43.8 (12.71) | 52.3 (11.41) |
| Pre-salbutamol FEV_1_/FVC, mean (SD) | 0.51 (0.08) | 0.60 (0.08) | 0.48 (0.09) | 0.58 (0.08) |
| Post-salbutamol FEV_1_/FVC, mean (SD) | 0.55 (0.08) | 0.63 (0.05) | 0.52 (0.09) | 0.62 (0.06) |
| Asthma exacerbations in previous year, n (%) |  |  |  |  |
| 1 | 335 (85.2) | 233 (79.3) | 334 (76.8) | 208 (77.3) |
| >1 | 58 (14.8) | 61 (20.7) | 101 (23.2) | 61 (22.7) |

Screening: PAL, airflow limitation, defined as post-salbutamol FEV_1_/FVC <LLN. On-treatment: AL+, airflow limitation, defined as all available post-randomisation 3 h post-dose FEV_1_/FVC <LLN; AL-, normalisation of airflow limitation, defined as at least one post-randomisation 3 h post-dose FEV_1_/FVC ≥LLN. LLN, lower limit of normal; BMI, body-mass index; ACQ, Asthma Control Questionnaire.

# Figures

Supplementary Figure 1. Patients in TRIMARAN and TRIGGER subgrouped by post-salbutamol LLN cut-point PAL status at screening (top panel), and the subgroup of patients with post-salbutamol LLN cut-point PAL at screening then subgrouped by on-treatment 3-h post-dose airflow limitation status during the studies.


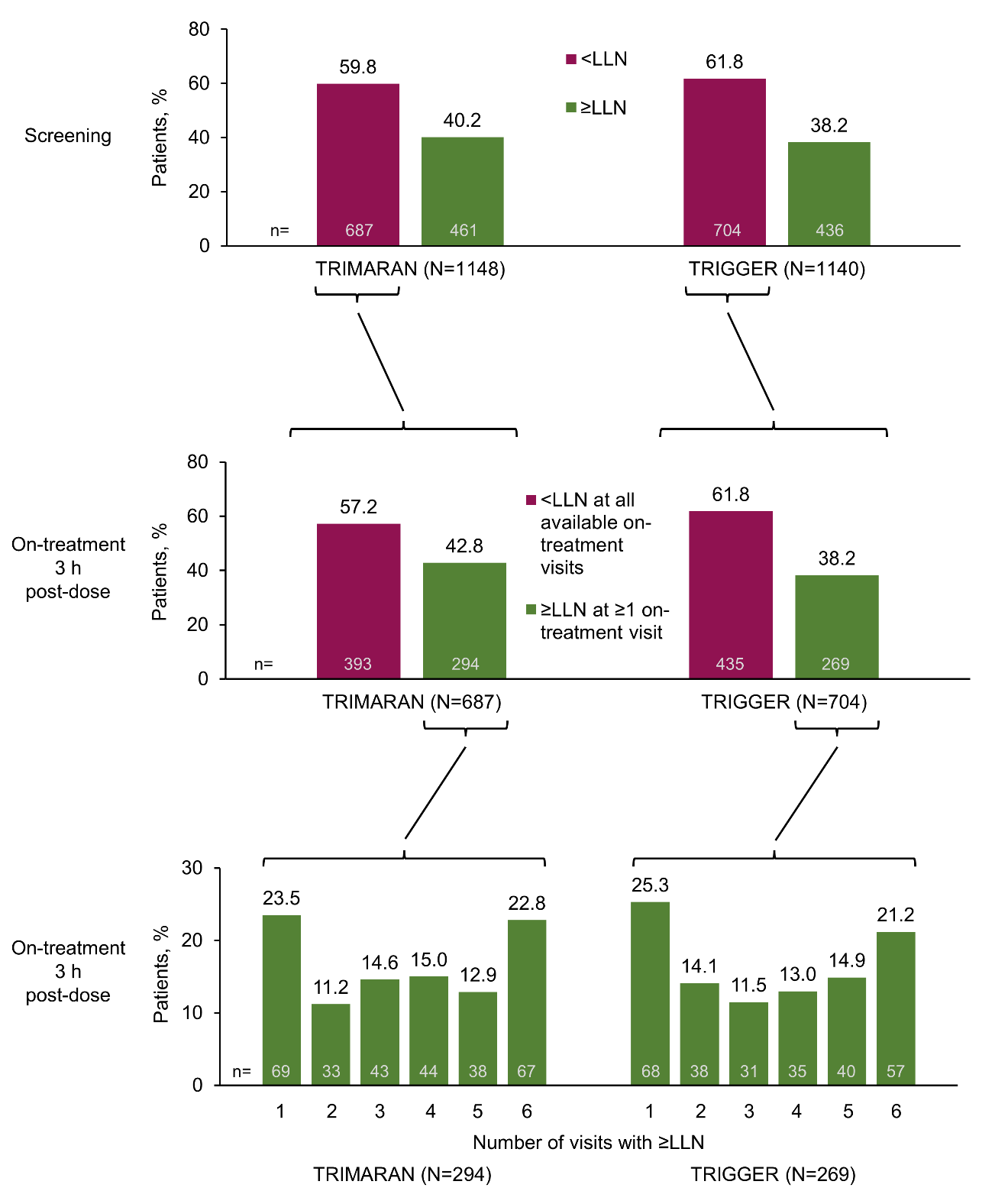


Screening: PAL, persistent airflow limitation, defined as post-salbutamol FEV_1_/FVC <LLN; No PAL, no persistent airflow limitation, defined as post-salbutamol FEV_1_/FVC ≥LLN. On-treatment: AL+, airflow limitation, defined as all available post-randomisation 3 h post-dose FEV_1_/FVC <LLN; AL-, normalisation of airflow limitation, defined as at least one post-randomisation 3 h post-dose FEV_1_/FVC ≥LLN. LLN, lower limit of normal.

Supplementary Figure 2. Adjusted rate of moderate/severe exacerbations in the subgroup of patients with post-salbutamol LLN cut-point PAL at screening, comparing patients by on-treatment 3-h post-dose LLN cut-point airflow limitation status.


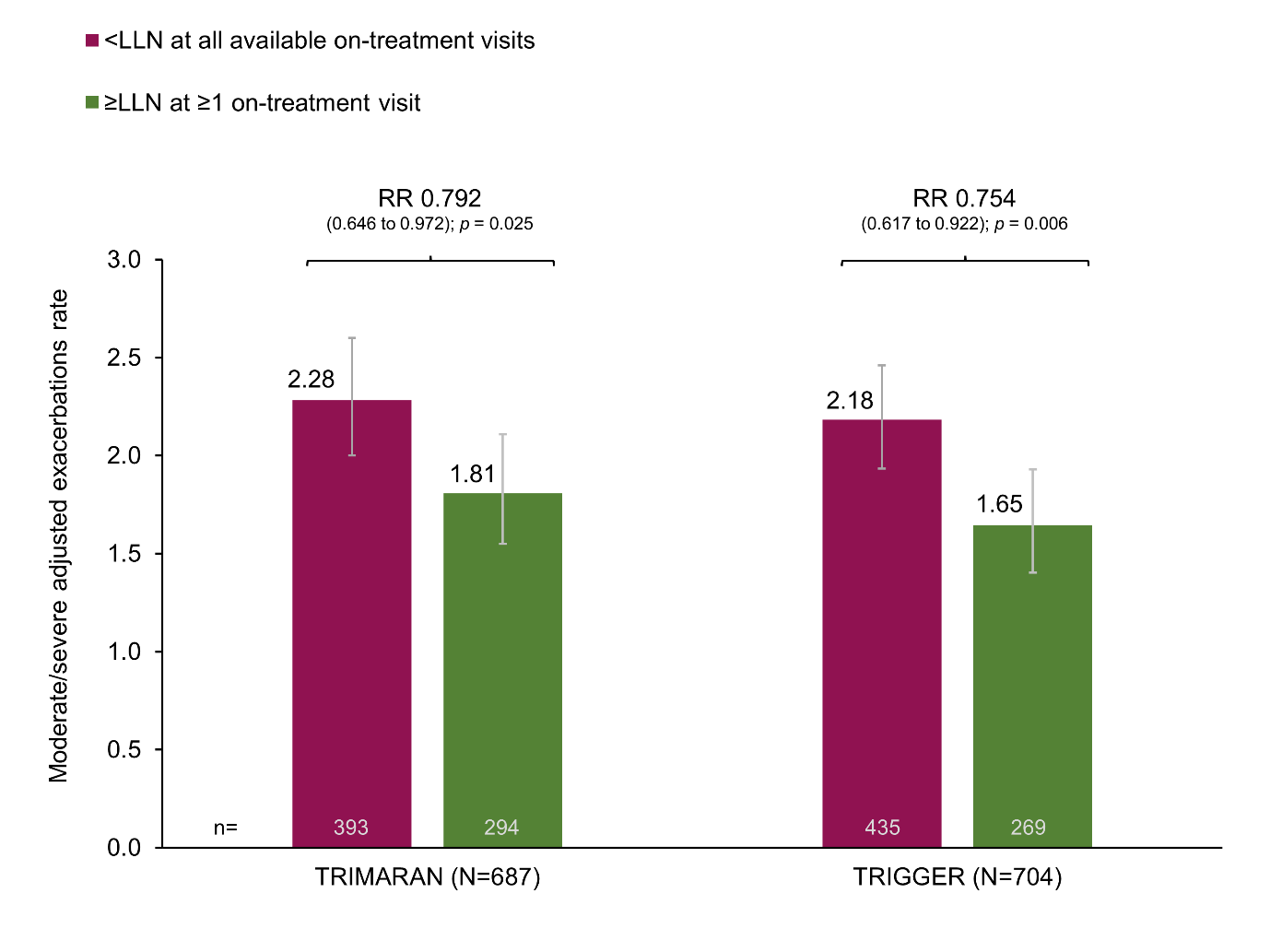


Data plotted are adjusted exacerbation rate and 95% confidence interval comparing patients without vs with on-treatment LLN-derived airflow limitation. <LLN, airflow limitation, defined as all available post-randomisation 3 h post-dose FEV_1_/FVC <LLN; ≥LLN, normalisation of airflow limitation, defined as at least one post-randomisation 3 h post-dose FEV_1_/FVC ≥LLN. PAL, persistent airflow limitation, defined as post-salbutamol FEV_1_/FVC <LLN at screening. RR, rate ratio (95% confidence interval). LLN, lower limit of normal.

Supplementary Figure 3. Relationship between study treatment received and normalisation of LLN cut-point airflow limitation.


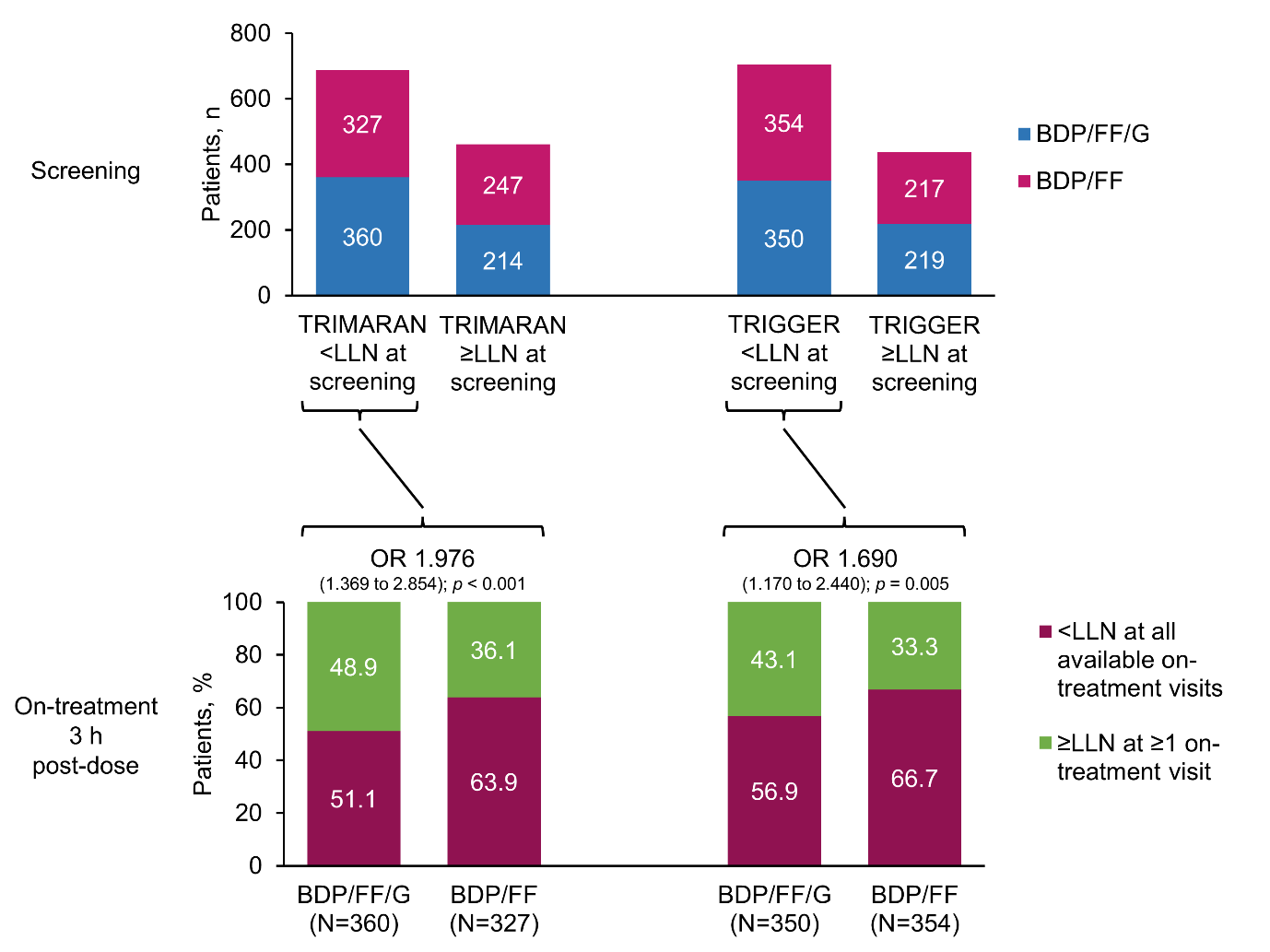


OR, odds ratio for the proportion of patients with FEV_1_/FVC≥LLN at ≥1 on-treatment visit, comparing BDP/FF/G vs BDP/FF. Screening: PAL, persistent airflow limitation, defined as post-salbutamol FEV_1_/FVC <LLN; No PAL-, no persistent airflow limitation, defined as post-salbutamol FEV_1_/FVC ≥0.7. On-treatment: <LLN, airflow limitation, defined as all available post-randomisation 3 h post-dose FEV_1_/FVC <LLN; ≥LLN, normalisation of airflow limitation, defined as at least one post-randomisation 3 h post-dose FEV_1_/FVC ≥LLN. BDP, beclometasone dipropionate; FF, formoterol fumarate; G, glycopyrronium; LLN, lower limit of normal.

Supplementary Figure 4. Adjusted rate of moderate/severe exacerbations in the subgroup of patients with post-salbutamol LLN cut-point PAL at screening, with patients subgrouped by on-treatment 3-h post-dose LLN cut-point airflow limitation status and by treatment.


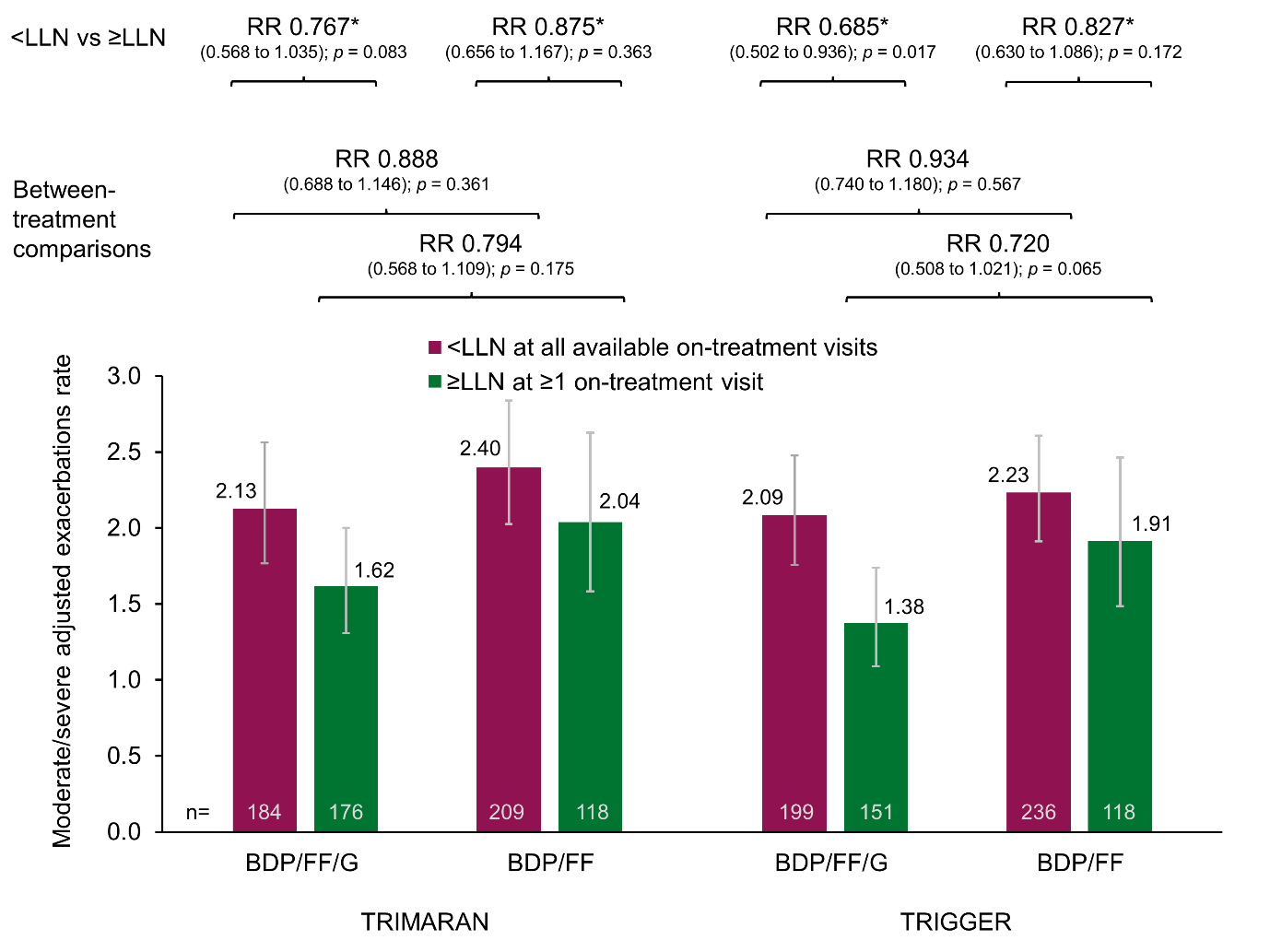


Data plotted are adjusted exacerbation rate and 95% confidence interval from the statistical model subgrouping patients by on-treatment airflow limitation and comparing BDP/FF/G vs BDP/FF (*the rates from the statistical model subgrouping patients by the treatment received and comparing patients without vs with on-treatment airflow limitation were 2.14, 1.64, 2.36, 2.06, 2.14, 1.47, 2.22 and 1.83). <LLN, airflow limitation, defined as all available post-randomisation 3 h post-dose FEV_1_/FVC <LLN; ≥LLN, normalisation of airflow limitation, defined as at least one post-randomisation 3 h post-dose FEV_1_/FVC ≥LLN. PAL, persistent airflow limitation, defined as post-salbutamol FEV_1_/FVC <LLN at screening. RR, rate ratio (95% confidence interval); BDP, beclometasone dipropionate; FF, formoterol fumarate; G, glycopyrronium; LLN, lower limit of normal..
